# Disease severity, treatment patterns, and quality of life in patients with moderate-to-severe psoriasis routinely managed with systemic treatment: results of the CRYSTAL observational study in Central and Eastern European countries

**DOI:** 10.3389/fimmu.2024.1410540

**Published:** 2024-05-23

**Authors:** Liisi Raam, Ilona Hartmane, Skaidra Valiukevičienė, Arfenya E. Karamova, Eniko Telegdy, Ivan Botev, Diana Marina, Simone Rubant, Teotonio Albuquerque, Maria Magdalena Constantin

**Affiliations:** ^1^ Department of Dermatology and Venereology, University of Tartu, Dermatology Clinic, Tartu University Hospital, Tartu, Estonia; ^2^ Department of Dermatology and Venereology, Faculty of Medicine, Riga Stradins University, Riga, Latvia; ^3^ Department of Skin and Venereal Diseases, Lithuanian University of Health Sciences (LSMU), Hospital of LSMU Kauno Klinikos, European Reference Network for Rare and Complex Diseases of the Skin (ERN-Skin) Member, Kaunas, Lithuania; ^4^ Dermatology Department, State Research Center of Dermatovenereology and Cosmetology, Moscow, Russia; ^5^ Dermatology Department, Markusovszky University Teaching Hospital, Szombathely, Hungary; ^6^ Ambulatory for Specialized Medical Care, Skin and Venereal Diseases, Sofia, Bulgaria; ^7^ AbbVie SRL Romania, Bucharest, Romania; ^8^ AbbVie Deutschland GmbH & Co. KG, Ludwigshafen, Germany; ^9^ AbbVie Portugal, Amadora, Portugal; ^10^ IInd Department of Dermatology, Colentina Clinical Hospital, Carol Davila University of Medicine and Pharmacy, Bucharest, Romania

**Keywords:** real-world, severity of illness index, patient-reported outcomes, psoriasis, systemic therapy

## Abstract

Psoriasis is a common, life-long skin disease with a significant negative health and societal impact. Data on rates of disease control and treatment strategies are lacking in Central and Eastern European countries. We aimed to describe the real-world disease severity, control, and treatment strategies for psoriasis in patients from Central and Eastern European countries. CRYSTAL (EUPAS36459) was a cross-sectional, retrospective study in adults (18–75 years) from Bulgaria, Estonia, Hungary, Latvia, Lithuania, Romania, and Russia. We enrolled patients with moderate-to-severe psoriasis receiving continuous systemic treatment for ≥24 weeks. We used the Psoriasis Area and Severity Index (PASI) to describe disease severity and the Dermatology Life Quality Index (DLQI) to assess quality of life (QoL) and collected other outcomes [psoriasis work productivity and activity impairment (WPAI-PSO), patient satisfaction] at enrollment. Analyses were descriptive. A total of 690 patients were included in the analyses. Median disease duration was 11.8 years. Current treatment was monotherapy for most patients (95.8%) with either biological (BIO group; 88.4%) or conventional (NON-BIO group; 7.4%) agents. Mean (± standard deviation) absolute PASI scores were 3.5 ± 5.7, 3.1 ± 5.3, and 6.6 ± 7.4 in the overall population, the BIO group, and the NON-BIO group, respectively. Among patients treated with monotherapy, absolute PASI scores ≤1, ≤3, and ≤5 were observed for 44.1%, 72.0%, and 82.6% of BIO patients and 21.6%, 33.3%, and 49.0% of NON-BIO patients. Mean DLQI total score was 3.3 ± 5.1; higher scores were noted for higher absolute PASI. The most impacted WPAI-PSO domain was presenteeism; for all domains, impact increased with increased absolute PASI. A total of 91.8% of BIO patients and 74.5% of NON-BIO patients were satisfied with the current treatment. We observed a better disease control in BIO than NON-BIO patients. However, around half of BIO patients did not reach clear skin status and reported an impact on QoL. An improvement in treatment strategies is still needed in Central and Eastern European countries to optimize outcomes of moderate-to-severe psoriasis.

## 1 Introduction

Psoriasis is a chronic, immune-mediated inflammatory skin disease affecting 2%–4% of the general population in Western countries (1, 2) and generating a high burden in terms of patients’ quality of life (QoL), comorbidity, and social costs (3–5). The Global Burden of Disease group has estimated more than 64.6 million psoriasis cases globally in 2017 (6) and 4,622,594 incident cases in 2019 (7). In Europe, country-specific prevalence estimates for psoriasis as diagnosed by physicians/dermatologists ranged from 0.51% to 2.36%, and an overall lower prevalence was reported in Central and Eastern European than in Western European countries (8). However, more recent reports indicate a higher burden of disease in Central and Eastern Europe: in Latvia, the estimated annual incidence between 2015 and 2020 was 2.1–2.2 cases per 1,000 person-years (9), and, in Romania, a prevalence of 4% was estimated between November 2018 and February 2019 (10).

The severity of psoriasis depends to a significant degree on the extension of lesions, which may range from a few scattered red, scaly plaques to involvement of almost the entire body surface, impacting severely the individual’s QoL. Several factors such as disease severity, gender, age, anatomical sites of lesion, comorbidity(ies), psychological distress and burden, and time needed for treatment have been associated with a reduced health-related QoL (HRQoL) (11). Most psoriasis patients also experience negative impact on work, emotions, and relationships (12).

Treatment effectiveness and convenience have been proposed as measures of patients’ satisfaction with therapy under real-world conditions (13), and treatment regimens are frequently adjusted in order to maximize effectiveness.

The growing variety of treatments have increased expectations to achieve a complete/almost complete resolution of disease symptoms, as observed in clinical trials (14). International guidelines (15–17) recently incorporated the Psoriasis Area and Severity Index (PASI) absolute scores (e.g., PASI ≤2) as treatment targets (in addition to relative scores), as they reflect the efficacy of a treatment regardless of disease severity at baseline. Moreover, absolute PASI is known to better correlate with the Dermatology Life Quality Index (DLQI) than relative PASI.

Biological therapy has shown good effectiveness in real-world settings (18) and greater efficacy and improvement in the patients’ HRQoL compared to conventional agents (19, 20). However, the use of biologics in psoriasis patients is not uniformly implemented in Central and Eastern Europe (21) and recent data on the type of treatment used in real-life settings are lacking (21, 22). The main objective of this study was to describe psoriasis severity, by absolute PASI scores, for patients from Central and Eastern European countries with moderate-to-severe psoriasis under systemic treatment in the clinical setting. We also aimed to describe treatment patterns, HRQoL, work and activity impairment, and treatment satisfaction in these patients.

## 2 Material and methods

### 2.1 Study design and participants

We conducted an epidemiological, multi-country, multicenter, cross-sectional, retrospective study between September 2020 and February 2021, in 29 hospital centers/clinics/practices (public or private) specialized in dermatology from seven Central and Eastern European countries: Bulgaria, Estonia, Hungary, Latvia, Lithuania, Romania, and Russia. The study involved a single visit where eligibility was assessed, informed consent was obtained, and study data were collected. All treatments were administered according to routine clinical practice. Study approvals were obtained from National and/or local Ethics Committees in all participating countries. The study was designed and conducted in accordance with the Declaration of Helsinki, the Good Pharmacoepidemiology Practices guidelines of the International Society for Pharmacoepidemiology, as well as local regulations.

This study included patients meeting all selection criteria who accepted to participate. Eligible patients were aged 18–75 years with confirmed diagnosis of moderate-to-severe chronic plaque–type psoriasis and treated with any approved systemic treatment for psoriasis (mono- or combination therapy) continuously for at least 24 weeks, who had absolute PASI assessed at the start of their current systemic treatment (including during a window between 30 days prior and 7 days after) and were expected to have absolute PASI assessment at enrollment (i.e., the study visit). Patients receiving treatment with any investigational intervention or who had received treatment within 1 month or 5 half-lives of the agent were not eligible.

The study is registered in The European Union electronic Register of Post-Authorization Studies (EUPAS36459).

### 2.2 Data collection

The data collected at the study baseline visit included medical history and baseline demographic and behavioral characteristics, including smoking habits. Disease severity by absolute PASI [calculated as described in Text S1 (23)], comorbidities, treatment for psoriasis, and patient-reported outcomes in terms of HRQoL [DLQI (24)], work productivity and activity impairment [WPAI (25)], and patient satisfaction with treatment were collected. Disease characteristics at psoriasis diagnosis, clinically relevant medical history (including psoriatic arthritis), past treatments for psoriasis, and information about the current treatment from its initiation until enrollment (i.e., date of initiation, starting dosage, and dosage intensifications) were also recorded. All data were entered into a password-protected, web-based electronic data capture system by the physician.

Patients completed the DLQI, EQ-5D-5L [including the EuroQol-visual analog scale (EQ-VAS)], and WPAI-PSO questionnaires (paper forms), and scores were calculated as described in **Supplementary Text S1**.

### 2.3 Statistical analysis

Sample size calculation was based on the primary endpoint. A sample size of 630 patients was estimated to produce a two-sided 95% confidence interval with a distance from the mean to limits equal to 0.078 for an estimated standard deviation (SD) of 1.0.

Statistical analyses were mainly descriptive and were performed on the full analysis set, including all eligible patients with available data. In addition, where indicated by the study objectives, analyses were also performed in the study subpopulations by current systemic treatment option and by absolute PASI score at the study visit, as applicable. Exploratory statistical tests were used only in the context of examining the correlation between HRQoL/DLQI/EQ-VAS and the absolute PASI score at the study visit, and the potential association of factors of interest with primary and secondary outcomes.

Continuous variables were examined with the Shapiro–Wilk test for normality. The correlation between continuous variables was evaluated by use of the Spearman’s ρ correlation coefficient. The effect of factors of interest on the primary outcome variable (absolute PASI score at the study visit) was assessed by linear regression models. The potential influence of confounding factors on the associations was examined through multivariable linear regression analysis. The following variables were entered in the initial step of the stepwise procedure based on minimization of the Akaike’s information criterion:

- For absolute PASI score at study visit: absolute PASI at the start of current treatment (or most recent assessment), comorbid psoriatic arthritis and/or spondylitis and/or enthesitis and/or dactylitis at start of current treatment, current systemic treatment with biological agents, disease duration at the start of current treatment, duration of current systemic treatment, gender, number of previous treatment courses with biological agents, physician-reported disease severity at the start of current treatment, positive family history of psoriasis, nail psoriasis at the start of current treatment, comorbidities diagnosed prior to the start of current treatment, and prior use of biological agent(s) before the start of current treatment. Gender was not identified as a confounder and thus was included in the initial step of the stepwise procedure. Patients’ age at the start of current treatment was identified as a confounder of the association between absolute PASI score at study visit and disease duration (years) at the start of current treatment (continuous); therefore, it was excluded from the stepwise process and was added in the final model.
- For PASI ≤1 achievement at study visit: absolute PASI at the start of current treatment (or most recent assessment), comorbid psoriatic arthritis and/or spondylitis and/or enthesitis and/or dactylitis at the start of current treatment, current systemic treatment with biologics, disease duration at the start of current treatment, duration of current systemic treatment, gender, number of previous treatment courses with biologic agents, patients’ age at the start of current treatment, physician-reported disease severity at the start of current treatment, positive family history of psoriasis, nail psoriasis at the start of current treatment, comorbidities diagnosed prior to the start of current treatment, and prior use of biologic agent(s) before the start of current treatment.

Variables that were examined in both continuous and categorical forms by univariable regression models were included in the multivariable model in the form corresponding to a lower p-value in the univariable analysis. Independent variables with a missing data rate exceeding 10% were not included in the multivariable analysis. All statistical tests were two-sided and were performed at a 0.05 significance level.

Analyses were performed using the SAS statistical software package.

## 3 Results

### 3.1 Patients, disease, and treatment characteristics

Of 694 enrolled patients, 690 were included in the full analysis set. For four patients, inclusion criteria were not met (“age between 18 and 75 years old” for two patients and “continuous systemic treatment for psoriasis for at least 24 weeks” for two patients).

The median age at enrollment was 49.7 [interquartile range (IQR), 39.4–60.2] years, and most patients were men (64.9%; 448). Nearly half (46.5%; 321) of patients had at least one comorbidity (**Table 1**), and 89 (13.0%) of 682 patients with available data had active psoriatic arthritis.

**Table 1:** Patient sociodemographic, anthropometric, lifestyle, and clinical characteristics at study visit (full analysis set, N = 690).

Characteristic	Value
Median (IQR) age, years	49.7 (39.4–60.2)
Male sex, n (%)	448 (64.9%)
Race, n (%)	
Caucasian	688 (99.7%)
African	1 (0.1%)
Not reported	1 (0.1%)
Place of residence, n (%)	
Urban	519 (75.2%)
Semi-urban	67 (9.7%)
Rural	104 (15.1%)
Education level	
No education	2 (0.3%)
1–6 years of education	10 (1.4%)
7–9 years of education	50 (7.2%)
10–12 years of education	309 (44.8%)
≥13 years of education	316 (45.8%)
Not reported	3 (0.4%)
Marital status, n (%)	
Married	505 (73.2%)
Single	108 (15.7%)
Divorced/Separated	40 (5.8%)
Widowed	26 (3.8%)
Not reported	11 (1.6%)
Employment status, n (%)	
Employed (paid employee or self-employed)	442 (64.1%)
Unemployed	83 (12.0%)
Retired	138 (20.0%)
Household duties	13 (1.9%)
Student	14 (2.0%)
Mean (SD) weight,^a^ kg	85.7 (17.9)
Mean (SD) height,^a^ cm	173.1 (9.7)
Mean (SD) BMI,^b^ kg/m^2^	28.6 (5.5)
Smoking status, n (%)	
Never smoked	363 (52.6%)
Occasional smoker	77 (11.2%)
Current smoker	157 (22.8%)
Former smoker	93 (13.5%)
Alcohol consumption in the previous month, n (%)	
None	297 (43.0%)
Occasional (1–2 units/week)	342 (49.6%)
Regular (>2 units/week)	49 (7.1%)
Not reported/unknown	2 (0.3%)
Clinically significant medical/surgical history and comorbidities, n (%)	321 (46.5%)
At least one past medical condition/disease/surgery	106 (15.4%)
At least one ongoing medical condition/comorbidity	283 (41.0%)
At least one medical condition/comorbidity with unknown status	1 (0.1%)

n (%), number (percentage) of patients in each category; N, total number of patients; BMI, body mass index; IQR, interquartile range; SD, standard deviation.

a Data were available for 689 patients.

b Data were available for 685 patients.

Median disease duration was 11.8 (IQR, 5.8–21.8) years (**Table 2**). At the current systemic treatment initiation, 95.2% (657) of patients had received at least one prior treatment, which had been discontinued at current systemic treatment initiation, with 552 (80.0%) and 625 (90.6%) patients having received systemic and non-systemic treatment, respectively. For the previous systemic treatment, the type of treatment received was conventional agents [73.9% (68.7% methotrexate)], phototherapy (62.0%), biological agents {27.1% [20% tumor necrosis factor (TNF) inhibitors, 6.7% interleukin-17 (IL-17) inhibitors, and 3.3% IL-12/23 inhibitors]}. The current systemic treatment had been received over a median period of 27.7 months. Most patients (95.8%) were receiving monotherapy with either biological (88.4%) or conventional (7.4%) agents, whereas 4.2% of patients were under a combination treatment (**Table 2**). During the current systemic treatment, 47.0% (324) of patients had received at least one concomitant topical treatment, which was ongoing at enrollment in the study for 29.4% (203) of patients (with keratolytic agents such as salicylic acid for 21.7% and corticosteroids for 14.6% of patients). Among patients receiving biological monotherapy, treatment was intensified (i.e., dose increased or time between doses decreased) in 3.1% (19) of patients (mainly due to insufficient response).

**Table 2:** Disease and treatment history and characteristics (full analysis set, N = 690).

Characteristic	Value
Median (IQR) age at psoriasis signs and symptoms onset, years	26.0 (17.7–39.2)
Median (IQR) age at plaque psoriasis diagnosis, years	32.9 (20.8–46.6)
Median time (IQR) from psoriasis signs and symptoms onset to plaque psoriasis diagnosis, years	1.0 (0.0–6.0)
Median time (IQR) from psoriasis signs and symptoms onset to study visit, years	18.7 (11.7–26.6)
Median time (IQR) from plaque psoriasis diagnosis to study visit, years	11.8 (5.8–21.8)
Psoriasis severity at initial diagnosis, n (%)	
Mild	172 (24.9%)
Moderate	250 (36.2%)
Severe	169 (24.5%)
Unknown	99 (14.3%)
Median (IQR) PASI score at initial psoriasis diagnosis^a^	18.2 (12.2–22.8)
Presence of psoriatic plaques, n (%)	570 (82.6%)
Upper extremities (excluding nails)	401 (70.4%)
Lower extremities	375 (65.8%)
Trunk (posterior/anterior)	254 (44.6%)
Head (including scalp, face, neck, and ears)	230 (40.4%)
Nail psoriasis	189 (33.2%)
Genitals/groin	26 (4.6%)
Intertriginous areas	25 (4.4%)
Positive family history of psoriasis	
Yes	207 (30.0%)
No	443 (64.2%)
Unknown	40 (5.8%)
Active psoriatic arthritis, and/or dactylitis, and/or spondylitis, and/or enthesitis, and/or nail psoriasis, n (%)	
Yes	251 (36.4%)
No	429 (62.2%)
Unknown	10 (1.4%)
History of psoriatic arthritis, n (%)	
Yes	216 (31.3%)
No	469 (68.0%)
Unknown	5 (0.7%)
Severe itching/pruritus over the past 7 days, n (%)	117 (17.0%)
Current systemic treatment for psoriasis, n (%)	
Monotherapy with biological agent	610 (88.4%)
TNF inhibitor	335 (48.6%)
IL-17 inhibitor	167 (24.2%)
IL-12/23 inhibitor	89 (12.9%)
IL-23 inhibitor	19 (2.8%)
Monotherapy with conventional agent	51 (7.4%)
Combination therapy	29 (4.2%)
TNF inhibitor + conventional agent	23 (3.3%)
IL-12/23 inhibitor + conventional agent	4 (0.6%)
IL-17 inhibitor + conventional agent	2 (0.3%)
Median (IQR) duration of current systemic treatment, months	27.7 (14.3–59.6)
Monotherapy	26.7 (14.3–57.8)
Biological agents	28.9 (15.0–61.0)
Conventional agents	15.5 (9.2–23.7)
Combination therapy	59.5 (41.5–98.2)
Median (IQR) duration of uninterrupted current systemic treatment, months	24.0 (12.0–58.0)
Monotherapy	24.0 (12.0–55.6)
Biological agents	24.2 (12.0–60.0)
Conventional agents	14.0 (9.0–21.0)
Combination therapy	53.0 (36.0–92.1)
Median (IQR) PASI score at the start of current systemic treatment	20.0 (14.0–25.0)
Monotherapy	20.0 (14.0–25.0)
Biological agents	20.0 (14.1–25.0)
Conventional agents	16.8 (12.0–24.4)
Combination therapy	19.7 (15.0–29.6)
Mean (SD) PASI score at study visit	3.5 (5.7)
Monotherapy	3.5 (5.8)
Biological agents	3.1 (5.4)
Conventional agents	8.7 (7.9)
Combination therapy	3.1 (4.7)
Median (IQR) PASI score at study visit	1.4 (0.4–4.2)
Monotherapy	1.4 (0.4–4.2)
Biological agents	1.2 (0.4–3.6)
Conventional agents	6.0 (1.7–13.5)
Combination therapy	1.6 (0.3–3.9)

n (%), number (percentage) of patients in each category; N, total number of patients; IQR, interquartile range; IL, interleukin; PASI, Psoriasis Area and Severity Index; SD, standard deviation; TNF, tumor necrosis factor.

Percentages are calculated relative to the number of patients with available data. The duration of treatment (months) was calculated as (Date of study visit − Date of treatment onset + 1)/30.42. All partial missing dates were imputed: (i) for start dates, if only the day or the month were missing, they were set as the first day of the month or the first month of the year, respectively; (ii) for end dates, the reverse was applied; (iii) if only the year was available, both the day and the month were imputed as described in (i) and (ii).

a Calculated for 209 patients with available data.

### 3.2 PASI scores

**Figure 1:**
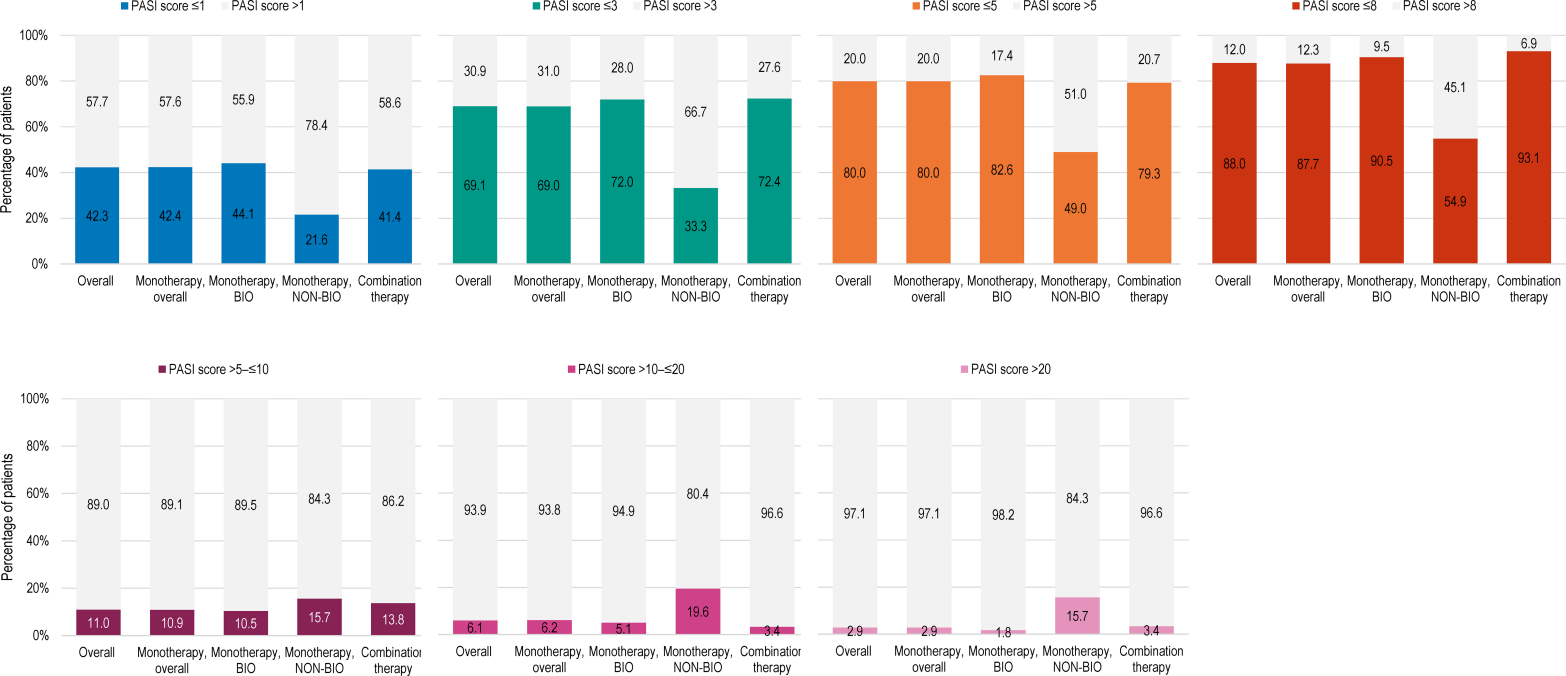
Distribution of patients by PASI score at study visit, in the overall population, and by current systemic treatment received (full analysis set). BIO, patients treated with biological agents; NON-BIO, patients treated with non-biological (conventional) agents; PASI, Psoriasis Area and Severity Index.

The mean (SD) absolute PASI score was 3.5 (5.7) (**Table 2**). The mean absolute PASI was 3.1 (5.3) and 6.6 (7.4) for biological agents and non-biological agents. The proportions of patients with absolute PASI scores ≤1, ≤3, and ≤5 were 42.3%, 69.1%, and 80.0%, respectively. An absolute PASI score <1 was achieved by 44.1% and 21.6% of patients for biologics (monotherapy) and non-biologics, respectively. Absolute PASI scores ≤1, ≤3, and ≤5 were observed for 44.1%, 72.0%, and 82.6% of patients receiving monotherapy with biological agents and 21.6%, 33.3%, and 49.0% of patients receiving monotherapy with conventional agents (**Figure 1**).

In multivariate analyses, lower absolute PASI scores at the start of current systemic treatment, current systemic treatment with biologics, higher disease duration at the start of current treatment, higher duration of current systemic treatment, and lower patient age at the start of current treatment were associated with decreased absolute PASI scores at the study visit. Lower patient age at the start of treatment, disease duration ≥10 years, current systemic treatment with biologics, absence of nail psoriasis, moderate psoriasis at the start of the current treatment, and female gender were associated with increased odds of reaching an absolute PASI score of ≤1 (**Table 3**).

**Table 3:** Results of multivariable linear regression analysis with factors of interest for absolute PASI score at study visit and for absolute PASI ≤1 achievement at study visit (N = 679).

Parameter	Effect of factor on the outcome^a^
Association with absolute PASI score at study visit	
Absolute PASI at the start of current treatment	Each one-unit increase in the absolute PASI score at the start of current treatment was found to be associated with a 0.09-unit increase in the absolute PASI score (95% CI: 0.05, 0.14; p < 0.001).
Current systemic treatment with biologics	Current systemic treatment with biologics versus with no biologics was found to be associated with a 4.96-unit decrease in the absolute PASI score (95% CI: −6.58, −3.33; p < 0.01).
Disease duration (years) at the start of current treatment	Each 1-year increase in disease duration at the start of current treatment was found to be associated with a 0.06-unit decrease in the absolute PASI score (95% CI: −0.09, −0.02; p = 0.003).
Duration (months) of current systemic treatment	Each 1-month increase in the duration of current systemic treatment was found to be associated with a 0.03-unit decrease in the absolute PASI score (95% CI: −0.04, −0.02; p < 0.01).
Patient age at the start of current treatment	Each 1-year increase in patient age at the start of current treatment was associated with a 0.05-unit increase in the absolute PASI score (95% CI: 0.01, 0.08; p < 0.005).
Number of previous treatment courses with biological agents	Not statistically significantly associated with the absolute PASI score (p = 0.185)
Nail psoriasis at the start of current treatment	Not statistically significantly associated with the absolute PASI score (p = 0.182)
Association with PASI ≤1 achievement at study visit	
Patient age at the start of current treatment	For each 1-year increase in patient age, the odds of achieving an absolute PASI score of ≤1 decreased by 1% (OR: 0.99; 95% CI: 0.98, 1.00; p = 0.048)
Disease duration at the start of current treatment	Patients with disease duration of ≥10 years at the start of current treatment had 1.6-fold higher odds of achieving an absolute PASI score of ≤1 than those with disease duration <10 years (OR: 1.60; 95% CI: 1.16, 2.21; p = 0.004)
Current systemic treatment with biologics	Patients treated with biologics had 3.0-fold higher odds of achieving an absolute PASI score of ≤1 compared with those currently treated with non-biologics (OR: 3.02; 95% CI: 1.49, 6.11; p = 0.002)
Nail psoriasis at the start of current treatment	Patients with nail psoriasis had 40% lower odds of achieving an absolute PASI score of ≤1 than those without (OR: 0.60; 95% CI: 0.43, 0.83; p = 0.002)
Physician-reported disease severity at the start of current treatment	Patients with severe psoriasis had 31% lower odds of achieving an absolute PASI score of ≤1 than those with moderate psoriasis (OR: 0.69; 95% CI: 0.50, 0.94; p = 0.021)
Gender	Male patients had 31% lower odds of achieving an absolute PASI score of ≤1 compared with female patients (OR: 0.69; 95% CI: 0.49, 0.96; p = 0.026)

N, total number of patients; BIO, patients treated with biological agents; CI, confidence interval; NON-BIO, patients treated with non-biological (conventional) agents; OR, odds ratio; PASI, Psoriasis Area and Severity Index.

a When all other factors are held constant.

### 3.3 Quality of life

**Figure 2:**
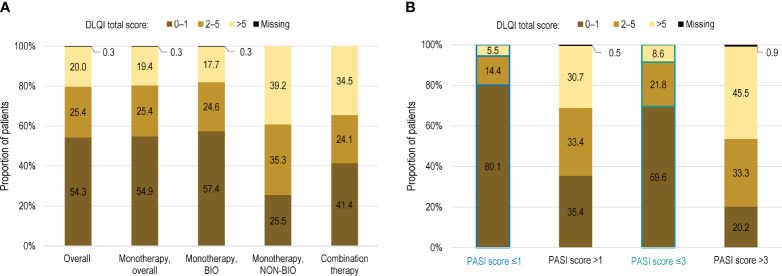
Distribution of patients by DLQI score in **(A)** the overall population, by current systemic treatment received; and **(B)** in the overall population with PASI score >1 and >3 (full analysis set). BIO, patients treated with biological agents; DLQI, Dermatology Life Quality Index; PASI, Psoriasis Area and Severity Index; NON-BIO, patients treated with non-biological (conventional) agents.

The mean (SD) DLQI total score in the overall population was 3.3 (5.1) (**Table 4**). Impairments in HRQoL, as shown by DLQI scores >5 were observed in 20.0% (138) of patients (**Figure 2A**). DLQI scores increased with higher PASI scores; among the patients with an absolute PASI scores >1 and >3, 30.7% (122/398) and 45.5% (97/213), respectively, had DLQI scores >5 (**Figure 2B**).

**Table 4:** Patient-reported outcomes at study visit, in the overall population, and by current systemic treatment received (full analysis set).

	Overall (N = 690)	Monotherapy, overall (N = 661)	Monotherapy, BIO (N = 610)	Monotherapy, NON-BIO (N = 51)	Combination therapy (N = 29)
Dermatology-specific health-related quality of life					
Patients with available data	688	659	608	51	29
Mean (SD) DLQI total score	3.3 (5.1)	3.2 (5.1)	2.9 (4.8)	7.1 (7.1)	4.0 (4.7)
General health-related quality of life					
Patients with available data	689	660	609	51	29
Proportion of patients with reported problems for each EQ-5D-5L dimension, n (%)					
Mobility	177 (25.7%)	163 (24.7%)	148 (24.3%)	15 (29.4%)	14 (48.3%)
Self-care	105 (15.2%)	96 (14.5%)	85 (14.0%)	11 (21.6%)	9 (31.0%)
Usual activities	138 (20.0%)	127 (19.2%)	107 (17.6%)	20 (39.2%)	11 (37.9%)
Pain/discomfort	256 (37.2%)	235 (35.6%)	208 (34.2%)	27 (52.9%)	21 (72.4%)
Anxiety/depression	202 (29.3%)	190 (28.8%)	170 (27.9%)	20 (39.2%)	12 (41.4%)
Mean (SD) EQ-5D-5L utility index score	0.9 (0.2)	0.9 (0.2)	0.9 (0.2)	0.8 (0.3)	0.8 (0.2)
Mean (SD) EQ-VAS score	78.8 (22.5)	79.1 (22.6)	80.0 (22.0)	68.2 (26.7)	72.3 (17.8)
Psoriasis-related work productivity loss and activity impairment					
Patients with available data	686	657	606	51	29
Patients employed	442	425	393	32	17
Mean (SD) WPAI-PSO domain scores					
Absenteeism	1.5 (9.2)	1.4 (9.3)	1.3 (8.8)	2.7 (14.1)	2.6 (7.3)
Presenteeism	7.8 (17.7)	7.5 (17.5)	6.6 (16.3)	18.1 (26.4)	14.4 (22.2)
Work productivity loss	8.3 (18.5)	8.0 (18.2)	7.1 (17.1)	18.3 (26.4)	16.3 (23.8)
Activity impairment	13.0 (23.0)	12.8 (22.9)	11.7 (21.9)	25.7 (29.2)	19.0 (26.0)
Patient satisfaction with control of psoriasis					
Patients with available data	686	657	606	51	29
Proportion of patients with scores on the single-item seven-point Likert-type scale, n (%)					
Satisfied	623 (90.3%)	598 (90.5%)	560 (91.8%)	38 (74.5%)	25 (86.2%)
Completely satisfied	408 (59.1%)	390 (59.0%)	374 (61.3%)	16 (31.4%)	18 (62.1%)
Mostly satisfied	167 (24.2%)	164 (24.8%)	148 (24.3%)	16 (31.4%)	3 (10.3%)
Somewhat satisfied	48 (7.0%)	44 (6.7%)	38 (6.2%)	6 (11.8%)	4 (13.8%)
Uncertain (either satisfied or dissatisfied)	23 (3.3%)	21 (3.2%)	17 (2.8%)	4 (7.8%)	2 (6.9%)
Dissatisfied	40 (5.8%)	38 (5.7%)	29 (4.8%)	9 (17.6%)	2 (6.9%)
Somewhat dissatisfied	10 (1.4%)	9 (1.4%)	7 (1.1%)	2 (3.9%)	1 (3.4%)
Mostly dissatisfied	14 (2.0%)	13 (2.0%)	8 (1.3%)	5 (9.8%)	1 (3.4%)
Completely dissatisfied	16 (2.3%)	16 (2.4%)	14 (2.3%)	2 (3.9%)	0 (0.0%)
Not answered	4 (0.6%)	4 (0.6%)	4 (0.7%)	0 (0.0%)	0 (0.0%)
Mean (SD) patient satisfaction score	1.8 (1.3)	1.8 (1.3)	1.7 (1.3)	2.7 (1.8)	1.9 (1.4)

n, number (percentage) of participants in each category; N, total number of patients; BIO, patients treated with biological agents; DLQI, Dermatology Life Quality Index; EQ-5D-5L, EuroQol 5-Dimensions 5-Levels; EQ-VAS, EuroQol-visual analog scale; NON-BIO, patients treated with non-biological (conventional) agents; SD, standard deviation; WPAI-PSO, Work Productivity and Activity Impairment Questionnaire for Psoriasis.

In the overall population, the correlation between the DLQI total score and the absolute PASI score was positive (Spearman ρ = 0.591, p < 0.001) (**Table 5**).

**Table 5:** Correlation of dermatology-specific and generic health-related quality of life with absolute PASI score as assessed by the Spearman’s rho correlation coefficient (full analysis set, N = 690).

	n	Spearman ρ coefficient	p-value for H0: ρ = 0	Level of correlation
DLQI total score	688	0.591	<0.001	Moderate positive correlation
EQ-5D-5L utility index score	689	−0.323	<0.001	Low negative correlation
EQ-VAS total score	689	−0.282	<0.001	Negligible correlation

n, number of patients with available data; N, total number of patients; DLQI, Dermatology Life Quality Index; EQ-5D-5L, EuroQol 5-Dimensions 5-Levels, EQ-VAS, EQ-VAS, EuroQol-visual analog scale; H0, null hypothesis.

The mean (SD) EQ-5D-5L utility score in the overall population was 0.9 (0.2). The most commonly reported negatively affected dimension was pain/discomfort, followed by anxiety/depression. The mean EQ-VAS score was 78.8 (22.5) (**Table 4**).

### 3.4 Work productivity and activity impairment

**Figure 3:**
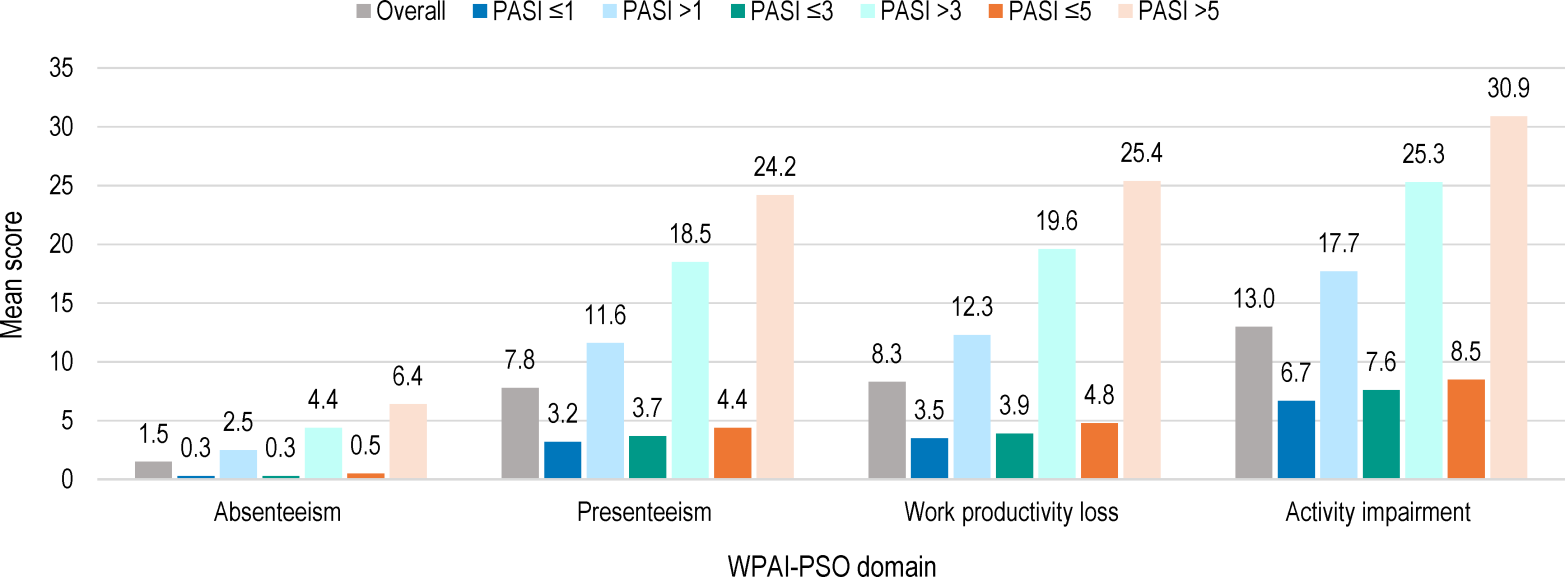
WPAI-PSO domain scores by absolute PASI scores at study visit (full analysis set). WPAI-PSO, Work Productivity and Activity Impairment Questionnaire for Psoriasis; PASI, Psoriasis Area and Severity Index. Note: Of the 690 patients in the full analysis set, 442 were employed.

Overall, 686 (99.4%) patients completed the WPAI-PSO questionnaire, of whom 442 (64.4%) were employed (**Table 4**). The domains with the highest impact of disease severity were presenteeism, followed by work productivity loss and activity impairment. For all domains, a higher impact was observed at higher PASI scores (**Figure 3**, **Supplementary Table S1**).

### 3.5 Patient satisfaction

Patient satisfaction questionnaires were completed by 99.4% of patients. In the overall population, most patients (90.3%) were satisfied with the overall control of the disease achieved with their current systemic treatment. The percentage of patients who were satisfied varied by systemic treatment: 91.8%, 74.5%, and 86.2% in patients receiving monotherapy with biological agents, monotherapy with conventional agents and combination therapy, respectively (**Table 4**).

## 4 Discussion

This large retrospective study is the first to provide extensive evidence on the control and treatment patterns of psoriasis in a large population of patients with moderate-to-severe disease, managed with systemic therapy in routine clinical settings across seven Central and Eastern European countries.

The median age at initial diagnosis was 32.9 years, most patients (64.9%) were men, and almost half (46.5%) had comorbidities. The characteristics of patients in our study compared well to those previously reported in another study conducted in 2008 in 913 patients from Central and Eastern Europe, considered as typical of a population of psoriasis patients (26).

We found that, after a median duration of 2.3 years of systemic treatment, mainly comprising biological monotherapy (in 88.4% of patients), more than 40% of participants with initial moderate-to-severe disease had absolute PASI scores ≤1, about two-thirds had scores ≤3, and 20% had not achieved scores ≤5. Absolute PASI scores can be an additional predictor for clinical response, which is more readily available in routine clinical settings than score improvements (27). Absolute PASI scores have been proposed as a measure of treatment goals, with values ≤2 or ≤3 indicating success and >5 indicating the need for a change in treatment, regardless of the baseline values (28). In our study, a higher proportion of patients with absolute PASI scores ≤3 (72.0% versus 33.3%) and a lower proportion with absolute scores >5 (17.4% versus 51.0%) were observed among the group treated with systemic biological therapy than those under conventional therapy. This finding is an indicator for better efficacy for systemic treatment using biological agents compared with conventional ones, in line with previous observations (29). In Europe, the recently updated EuroGuiDerm guidelines recommend initiation of systemic treatment for moderate-to-severe cases of psoriasis, using conventional agents as first-line and biological agents in the case of inadequate response, contraindications, or lack of tolerance to the conventional systemic treatment. However, initiation with biologics is recommended as a first-line treatment for severe psoriasis, when lack of success is anticipated with use of conventional agents (16). In contrast, French and British guidelines do not include biologics as first-line therapy, only as an option in case of lack of response to at least two conventional systemic therapies or in case of intolerance/contraindications (15, 17). The use of biologics can vary from one country to another and is impacted not only by national practices but also by criteria for coverage or access to specific agents. In contrast with previous reports from Central and Eastern European countries (21), our study indicated a wide use of biological therapy among patients with moderate-to-severe psoriasis, in line with current European recommendations (16).

Almost half of patients in our study had a DLQI score >1 and 20% of patients had a score >5. We observed a higher impact of the disease on HRQoL in patients treated with conventional agents than those treated with biological therapy. However, even among the latter, an impact on the QoL was reported by >40% of patients. In addition, a DLQI score >2 was still noted in around 20% of patients with absolute PASI score <1. This moderate impact of psoriasis is expected in the study population, comprising patients treated for at least 24 weeks, but still confirms the negative effect of the disease on the patient’s QoL. In a recent survey conducted in adult patients from 20 countries worldwide, 54% reported a very large to extremely large impact of psoriasis on their HRQoL (12), as indicated by DLQI scores ≥10. In a previous study conducted in Russia, psoriasis was shown to have a negative impact on patients’ HRQoL (with a reported mean DLQI score of 7.1) and work productivity (dropping by 33.2%), which increased further with disease severity (30). In a survey conducted in Romania, 35.7% of patients indicated that the disease affected their relationship with family and friends and 46.1% reported reduced social comfort in public places (31). We also established a moderate positive correlation between the DLQI and disease severity as indicated by PASI, in line with previous findings (32). Nevertheless, the effect of psoriasis on the QoL and the patient’s perception on achieving a therapeutic goal should be considered in a broader context, beyond a single point in time. Thus, the concept of cumulative life course impairment was previously proposed to express the ongoing, accumulated impact of psoriasis and its associated stigmatization and physical and psychological comorbidities over a patient’s life course (33), potentially preventing psoriasis patients from realizing their full life potential (34).

In our study, WPAI-PSO scores were low in the overall population, driven by the group of patients under biological systemic treatment, for which a positive impact on work productivity has been established (35–37). The patients’ satisfaction with the current systemic treatment was also higher in patients receiving biological agents compared to those taking conventional agents, with 91.8% versus 74.5% of responders being satisfied with their evolution, even among patients on biological therapy with PASI scores >5.

While our study helps building recent real-world evidence across Central and Eastern European countries about psoriasis control in patients with moderate-to-severe disease, the cross-sectional and retrospective design is associated with inherent limitations. We attempted to minimize patient selection bias by enrolling all consecutive patients reporting to study sites for visits and patient recall bias by using patient-reported outcomes that employ short or no recall period. The study was conducted during the COVID-19 pandemic; thus, the patients’ mental health and perceived QoL may have been adversely impacted, hindering direct comparisons with other studies. In addition, the results may have been influenced by differences between countries on multiple levels. National criteria for disease severity, reimbursement rules, and treatment guidelines differ from country to country (21), and this may have impacted the generalizability of our results. The use of the reimbursed treatment may have limited timely achievement of an adequate PASI score. For instance, in Latvia, biologic therapy is fully reimbursed only if initiated with adalimumab and the clinical effect must be observed for ≥16 weeks (38), thus potentially limiting the possibility of reaching the target PASI score faster by switching to a more effective biologic therapy.

## 5 Conclusion

A large proportion of patients with moderate-to-severe psoriasis are treated with bio-logical systemic therapy in Central and Eastern Europe and show low absolute PASI scores after at least 24 weeks of continuous treatment and an overall good satisfaction with their evolution. However, around half of patients with biological treatment did not reach clear skin status and reported an impact of the disease on the QoL, indicating that improvement in treatment strategies is still needed in Central and Eastern European countries to optimize the outcomes of patients with moderate-to-severe psoriasis.

## Data availability statement

The raw data supporting the conclusions of this article will be made available by the authors, without undue reservation.

## Ethics statement

The studies involving humans were approved by Bulgaria – Bulgarian Drug Agency approval number 28037/10.07.2020 (no requirement for explicit EC approval in observational studies at the time of study start), Estonia - Research Ethics Committee of the National Institute for Health Development (approval no. 430/27-Aug-2020), Ethics Committee of Medical Research Council of Medical Research Council (“ETT TUKEB”, approval no. IV/3394-7/2020/EKU/03-Jun-2020), Latvia - The Rīga Stradiņš University Research Ethics Committee (approval no. 280520-2L/28-May-2020), Lithuania – Lithuanian Bioethics Committee (approval no. L20-4/1 and L20-4/2/07-Jul-2020), Romania - National Bioethics Committee of Medicines and Medical Devices Romania (approval no. 9SNI/09-Jul-2020) and Russia - Independent Interdisciplinary Committee on Ethical Review of Clinical Studies (approval following the protocol meeting minutes no. 12/03-Jul-2020). The studies were conducted in accordance with the local legislation and institutional requirements. The participants provided their written informed consent to participate in this study.

## Author contributions

LR: Investigation, Validation, Writing – original draft, Writing – review & editing. IH: Investigation, Validation, Writing – original draft, Writing – review & editing. SV: Investigation, Validation, Writing – original draft, Writing – review & editing. AK: Investigation, Validation, Writing – original draft, Writing – review & editing. ET: Investigation, Validation, Writing – original draft, Writing – review & editing. IB: Investigation, Validation, Writing – original draft, Writing – review & editing. DM: Conceptualization, Data curation, Methodology, Project administration, Resources, Supervision, Writing – original draft, Writing – review & editing. SR: Conceptualization, Data curation, Methodology, Project administration, Resources, Supervision, Writing – original draft, Writing – review & editing. TA: Conceptualization, Data curation, Methodology, Project administration, Resources, Supervision, Writing – original draft, Writing – review & editing. MC: Investigation, Validation, Writing – original draft, Writing – review & editing.
