# Supplementary material for: Disease severity, treatment patterns, and quality of life in patients with moderate-to-severe psoriasis routinely managed with systemic treatment: results of the CRYSTAL observational study in Central and Eastern European countries
Source: Front Immunol. 2024 May 23;15:1410540. doi: 10.3389/fimmu.2024.1410540 (PMC11153796; doi:10.3389/fimmu.2024.1410540)
Supplement: Supplementary file 1 [file Table_1.docx]

Supplementary Material

# Supplementary Text S1. Scoring of physician assessments and patient-reported outcomes

## Psoriasis Area and Severity Index (PASI) component scores

PASI combines the assessment of lesion severity and the affected area into a total score ranging from 0 (no disease) to 72 (maximal disease). PASI measures the intensity of redness, thickness and scaliness of the lesions (each graded as 0 [none], 1 [mild], 2 [moderate], 3 [severe], 4 [very severe]) weighted by the percentage area of involvement (graded as 0 [0%], 1 [1–9%], 2 [10–29%], 3 [30–49%], 4 [50–69%], 5 [70–89%] or 6 [90–100%]) in each of the 4 regions (components) of the patient: head and neck, upper extremities, trunk, and lower extremities (1).

The PASI score was calculated as follows for each of the 4 PASI components:

- head and neck: A_1_*B_1_*0.1
- upper extremities: A_2_*B_2_*0.2
- trunk: A_3_*B_3_*0.3
- lower extremities: A_4_*B_4_*0.4

where A_i_ is the sum of the 3 intensity scores and B_i_ is the affected area score for each body region.

The total score was calculated as the sum of the 4 component scores.

## Dermatology Life Quality Index (DLQI)

DLQI consists of a set of 10 items/question (2). Each item is scored on a 4-point scale and a total score of 0–30 is calculated by summing all item scores. All items except for item 7 is scored as 0 (not at all/not relevant), 1 (a little), 2 (a lot), 3 (very much). Item 7 is scored as: 0 (no and a second half of not at all, no with an incomplete second half or not relevant), 1 (no and a second half of a little), 2 (no and a second half of a lot), 3 (yes).

The DLQI can be analyzed under 6 domain scores:

- symptoms and feelings: sum of scores for items/questions 1 and 2
- daily activities: sum of scores for items/questions 3 and 4
- leisure: sum of scores for items/questions 5 and 6
- work and school: score for item/question 7
- personal relationships: sum of scores for items/questions 8 and 9
- treatment: score for item/question 10.

If 2 or more questions were not answered, the total score was not calculated. When calculating domain scores, if the answer to one question was not answered, the score for that domain was not calculated.

## EuroQol 5-Dimensions 5-Levels (EQ-5D-5L)

The EQ-5D is a self-completed measure of the patient’s health-related quality of life and consists of the EQ-5D descriptive system and the EuroQol-visual analogue scale (EQ-VAS). The EQ-5D-5L descriptive system grades each of 5dimensions (mobility, self-care, usual activities, pain/discomfort and anxiety/depression) across 5 levels: 1 (no problems), 2 (slight problems), 3 (moderate problems),4 (severe problems) and 5 (extreme problems).

For the calculation of the EQ-5D utility index score, each patient’s responses were assigned to a health state, based on value sets provided by the trial sponsor.

## Work Productivity and Activity Impairment Questionnaire for Psoriasis (WPAI-PSO)

WPAI-PSO is a self- or interviewer-administered questionnaire used to assess productivity at work and activity impairment due to a specific health problem (3). WPAI-PSO evaluates 4 main outcomes through 6 questions:

- absenteeism (percent work time missed due to problem for those currently employed): Q2/(Q2+Q4)*100
- presenteeism (percent impairment while working due to problem for those currently employed and who actually worked in the 7 days prior to the administration of the questionnaire): Q5/10*100
- work productivity loss (percent overall work impairment due to problem for those currently employed): {Q2/(Q2+Q4) + [(1 – (Q2/Q2+Q4))*(Q5/10)]}*100
- activity impairment (percent activity impairment due to problem for all responders): Q6/10*100.

For those who missed work and did not actually work in the past 7 days prior to the administration of the questionnaire, the percent overall work impairment due to health was equal to the percent work time missed due to health.

## Patient satisfaction

The patient’s satisfaction with the overall control of psoriasis achieved with the current systemic treatment was measured using a single-item 7-point Likert-type scale ranging from satisfied (completely satisfied, mostly satisfied, somewhat satisfied), uncertain (either satisfied or dissatisfied) to dissatisfied (somewhat dissatisfied, mostly dissatisfied, completely dissatisfied).

## References:

1. Armstrong AW, Parsi K, Schupp CW, Mease PJ, Duffin KC. Standardizing training for psoriasis measures: effectiveness of an online training video on Psoriasis Area and Severity Index assessment by physician and patient raters *JAMA Dermatol* (2013) 149(5):577-82. doi:10.1001/jamadermatol.2013.1083

2. Finlay AY, Khan GK. Dermatology Life Quality Index (DLQI)--a simple practical measure for routine clinical use *Clin Exp Dermatol* (1994) 19(3):210-6. doi:10.1111/j.1365-2230.1994.tb01167.x

3. Reilly Associates. Work Productivity and Activity Impairment Questionnaire (WPAI) Scoring [homepage on the Internet]. 2002. [cited 2023 August 21]. Available from: <http://www.reillyassociates.net/WPAI_Scoring.html>.

# Supplementary Table S1. WPAI-PSO domain scores by absolute PASI scores at study visit (full analysis set)

|  | n | n_miss_ | Mean ± SD | Median | IQR | Min–Max |
| --- | --- | --- | --- | --- | --- | --- |
| Subpopulation with PASI ≤1 at study visit (N=292; N_employed_=199) | | | | | | |
| Absenteeism | 197 | 2 | 0.3 ± 1.8 | 0.0 | 0–0 | 0–16.7 |
| Presenteeism | 197 | 2 | 3.2 ± 12.6 | 0.0 | 0–0 | 0–100 |
| Work productivity loss | 197 | 2 | 3.5 ± 12.9 | 0.0 | 0–0 | 0–100 |
| Activity impairment | 291 | 1 | 6.7 ± 19.6 | 0.0 | 0–0 | 0–100 |
| Subpopulation with PASI >1 at study visit (N=398; N_employed_=243) | | | | | | |
| Absenteeism | 238 | 5 | 2.5 ± 12.3 | 0.0 | 0–0 | 0–100 |
| Presenteeism | 236 | 7 | 11.6 ± 20.3 | 0.0 | 0–20 | 0–100 |
| Work productivity loss | 236 | 7 | 12.3 ± 21.3 | 0.0 | 0–20 | 0–100 |
| Activity impairment | 395 | 3 | 17.7 ± 24.3 | 10.0 | 0–30 | 0–100 |
| Subpopulation with PASI ≤3 at study visit (N=477; N_employed_=316) | | | | | | |
| Absenteeism | 313 | 3 | 0.3 ± 2.1 | 0.0 | 0–0 | 0–25 |
| Presenteeism | 313 | 3 | 3.7 ± 11.7 | 0.0 | 0–0 | 0–100 |
| Work productivity loss | 313 | 3 | 3.9 ± 12.1 | 0.0 | 0–0 | 0–100 |
| Activity impairment | 475 | 2 | 7.6 ± 19.1 | 0.0 | 0–0 | 0–100 |
| Subpopulation with PASI >3 at study visit (N=213; N_employed_=126) | | | | | | |
| Absenteeism | 122 | 4 | 4.4 ± 16.8 | 0.0 | 0–0 | 0–100 |
| Presenteeism | 120 | 6 | 18.5 ± 24.9 | 10.0 | 0–25 | 0–100 |
| Work productivity loss | 120 | 6 | 19.6 ± 26 | 10.0 | 0–30 | 0–100 |
| Activity impairment | 211 | 2 | 25.3 ± 26.2 | 20.0 | 0–40 | 0–100 |
| Subpopulation with PASI ≤5 at study visit (N=552; N_employed_=364) | | | | | | |
| Absenteeism | 360 | 4 | 0.5 ± 2.8 | 0.0 | 0–0 | 0–28.6 |
| Presenteeism | 360 | 4 | 4.4 ± 12.5 | 0.0 | 0–0 | 0–100 |
| Work productivity loss | 360 | 4 | 4.8 ± 13.1 | 0.0 | 0–0 | 0–100 |
| Activity impairment | 548 | 4 | 8.5 ± 19.3 | 0.0 | 0–10 | 0–100 |
| Subpopulation with PASI >5 at study visit (N=138; N_employed_=78) | | | | | | |
| Absenteeism | 75 | 3 | 6.4 ± 20.8 | 0.0 | 0–0 | 0–100 |
| Presenteeism | 73 | 5 | 24.2 ± 27.8 | 20.0 | 0–30 | 0–100 |
| Work productivity loss | 73 | 5 | 25.4 ± 28.9 | 20.0 | 0–33.3 | 0–100 |
| Activity impairment | 138 | - | 30.9 ± 27.6 | 25.0 | 10–50 | 0–100 |

WPAI-PSO, Work Productivity and Activity Impairment Questionnaire for Psoriasis; PASI, Psoriasis Area and Severity Index; n, number of patients with available data for each category and domain; n_miss_, number of patients with data missing; SD, standard deviation; IQR, interquartile range; min, minimum; max, maximum; N, total number of patients; N_employed_, patients employed at study visit.
